# Alerting the illusion of smoking improves quality of life in Chinese male cancer survivors

**DOI:** 10.1002/cam4.1999

**Published:** 2019-02-08

**Authors:** Juan Xia, Peng Wu, Jiwei Wang, Jinming Yu

**Affiliations:** ^1^ Institute of Clinical Epidemiology, Key Laboratory of Public Health Safety, Ministry of Education, Key Lab of Health Technology Assessment of Ministry of Health, School of Public Health Fudan University Shanghai China

**Keywords:** cancer survivors, quality of life, smoking status, tobacco

## Abstract

**Objective:**

To examine the association between smoking status and quality of life (QOL) among cancer survivors in China.

**Methods:**

A cross‐sectional study was performed in 2725 male cancer survivors who were members of Cancer Rehabilitation Club and completed the questionnaires in 2013. Using linear regression models adjusted for confounders, we measured the association between QOL and former smokers as well as current (occasional, <10 cigarettes/day, and ≥ 10 cigarettes/day) smokers compared with never smokers.

**Results:**

Current smokers were reported to have higher scores in social, role, cognitive functioning, and had lower scores in nausea/vomiting, pain, dyspnea, and insomnia (*P* < 0.05). Former smokers had higher global health status and experienced less appetite loss and constipation (*P* < 0.05). Compared with never smokers, those former smokers and current smokers had significantly high scores on the global health status, social functioning, role functioning, and cognitive functioning (*P* < 0.05). And they had lower scores in some aspects of symptom scale (*P* < 0.05). Considering the dose of smoking, the scores were increased in functional subscales and decreased in symptom subscales with the increase of tobacco use, though few variables had statistical significance. As for smoking cessation, the proportion of lung cancer survivors who quit smoking was higher than that of other types of cancer survivors.

**Conclusion:**

Our study suggested the possibility that in China, where smoking prevalence is still high, continued smoking was associated with high QOL scores. The phenomenon may be obscured by some potential reasons, including subjectivity of questionnaire, special substances of cigarettes, Chinese unique culture of tobacco use, and much more. The results reminded researchers and clinicians some underlying situations among smokers in China and prompted a strong call for the implementation of a comprehensive tobacco‐control policy and specific public health educational strategies among not only lung cancer survivors but other types of cancers survivors.

## 1 INTRODUCTION

Cancer is the leading cause of death since 2010 and is a major public health problem in China currently.^1^ Previous studies revealed that approximately 4 291 000 Chinese became newly developed cancer cases and about 2 814 000 patients died from cancer in 2015, corresponding to over 7500 cancer deaths on average per day.^2^ A cancer survivor is any person who is “living with or beyond cancer,” namely someone who has completed initial cancer management and has no apparent evidence of active disease; or is living with progressive disease and may be receiving cancer treatment, but is not in the terminal phases of illness; or has had cancer in the former.^3^ Owing to the medical technology improving and population aging, the survival time was prolonged in cancer patients. It was estimated that, for all cancers combined, 36.9% of cancer patients in China would survive at least 5 years after diagnosis around 2015,^2^ which resulted in more attention paid to lifetime and quality of life (QOL) for cancer survivors. It was also estimated that 5%‐15% of cancer patients develop a subsequent primary cancer.^4^ There was evidence that the risk of subsequent primary cancer among cancer survivors was strongly associated with smoking behaviors.^5^, ^6^

Tobacco use is the essential preventable cause of premature death around the world with an estimated 30% of all kinds of cancer deaths attributed to smoking.^7^ The tobacco smoke, which contained more than 4000 known carcinogenic substances, is a risk factor responsible for many kinds of cancers,^8^ including cancers of lung,^9^ pancreatic,^10^ bladder,^11^ head, and neck^12^ and so forth. Also, multiple studies indicated that smoking may reduce the effectiveness of cancer therapies and increase the incidence of secondary cancer.^13^, ^14^ The dangers of smoking and the beneficial effects of smoking cessation on health were well‐established.^15^ However, less is known about how varied smoking status or smoking dose affects QOL.

QOL is an individual's perception of their life, which influenced by the atmosphere of culture and value systems that they lived, as well as one's standards and expectation.^16^ It is not only an essential outcome indicator in clinic and research, but also an important factor influencing mortality.^17^, ^18^ A great deal of researches showed that smoking had a negative impact upon QOL of cancer survivors in foreign countries.^13^, ^19^, ^20^ Regarding some special features in China, we launched this research through comparing QOL among never smokers, former smokers, occasional smokers, smokers with less than 10 cigarettes per day, and smokers with not less than 10 cigarettes per day to discover or evaluate the influence of tobacco use on QOL among Chinese male cancer survivors.

## 2 METHODS

### 2.1 Design and recruitment

A cross‐sectional study was conducted among cancer survivors from April to July 2013 in Shanghai Cancer Rehabilitation Club, which recruited from communities and hospitals through extensive channels covering all 17 counties of Shanghai. The inclusion criteria for study enrollment were as shown below: (a) at least 16 year‐old; (b) have pathological diagnosis of cancer; (c) able to independently participate in the cancer rehabilitation club; (d) willingness to provide written informed consent; (e) no cognitive impairment.

In total 10 023 cancer survivors registered in this club met the above criteria. Four hundred and fifty‐four failed to participate in the survey and they were unwilling to join causing no time, poor health condition, or other reasons. About 9569 members were participated this survey and the response rate was 95.4%. Questionnaires were determined as incomplete when one‐third or more questions were missing. Ultimately, it had 9125 valid questionnaires and the valid rate was 95.4%. Male and female cancer survivors were accounted for 29.9% (2725) and 70.1% (6400), respectively.

Of the total sample, the data for the analyses on the smoking status and QOL were available for 2725 male cancer survivors, since there were few female smokers in China. The information leaflets about content and purpose of this study and written informed consent forms were obtained from patients who met the inclusion criteria ahead of the investigation. Questionnaires were collected through face‐to‐face interviews who were students from the School of Public Health, Fudan University, either self‐administered by literate participants or completed with the help of well‐trained field workers to fill all the forms out lest the questionnaires have missing information. Once missing information was found, fieldworkers were responsible to reconfirm and ask participants to complete the questions. All data were entered using Epidata 3.0 software by double entry method.

## 3 MEASURES

### 3.1 Demographic and clinical characteristics

The demographic and clinical characteristics included age, gender, height, weight, marital status, residency, education level, work status, financial status, religion, medical insurance, coexisting illness, data of diagnosis, the primary cancer, therapeutic method, cost of treatment, the situation of recovery, time of taking part in activities, and more detailed information.

### 3.2 Smoking status

Smoking status was assessed based on response of the following questions, “Do you smoke at least 100 cigarettes in your lifetime?”(Yes/No). Those who answered “No” should jump directly to the next part (physical activities). The rest were further questions about present smoking status (no smoking, occasional smoking, smoking with less than 10 cigarettes per day, and smoking with no less than 10 cigarettes per day). Those who answered “no smoking” needed to report the specific smoking cessation time, and their inclination to quit tobacco and made practical action last for 1 day and above before or after the diagnosis of cancer.

The definition of smoking status classification was listed as followed. Never smokers were respondents who did not smoke during the survey as well as in the past. Former smokers were respondents who did not smoke at the time of the survey but smoked in the past. Current smokers were respondents who smoked during the survey, including daily smokers and occasional smokers. Occasional smokers were respondents who smoked occasionally during the survey.

### 3.3 Quality of life

EORTC QLO‐C30 (European Organization for the Research and Treatment of Cancer Quality of life Questionnaire‐Core 30, version 3.0)^21^ with well‐documented validity and reliability in various populations was translated and simplified in assessing health outcome of Chinese breast cancer survivors.^22^, ^23^

The questionnaire consisted of 30 items, including five functional subscales (physical, role, cognitive, emotional, and social); three symptom subscales (fatigue, nausea/vomiting, and pain); one global health status scale/QoL; and six single measurement items (dyspnea, insomnia, appetite loss, constipation, diarrhea, and financial difficulties). Each item had four options in the first 28 questions, listed as “not at all,” “a little,” “quite,” “very much,” and the rest items had seven degrees. Raw score needed to be adjusted into 0‐100‐point scales,^24^, ^25^ and higher mean scores in functional and global health scales/QOL indicated better functioning, whereas higher mean scores in the rest represented more severe symptoms.^25^

### 3.4 Ethical consideration

This study was approved by Medical Research Ethics Committee of the school of public health, Fudan University (The international registry NO. IRB00002408 & FWA00002399).

### 3.5 Data analysis

All statistical analyses were performed using the Statistical Analysis System (SAS, Version 9.4). Participants’ characteristics and QOL were summarized by percentages for categorical variables and mean (standard deviation) or median (Interquartile range) for continuous variables. Differences in means for continuous variables were compared using analysis of variance (ANOVA), and differences in proportions were tested by chi‐square test. After controlling confounders (including age, BMI, marital status, residency, education level, financial status, chronic diseases, and treatment status), standard least squares regression was used to evaluate the independent association between smoking status and QOL. Bonferroni test was applied to analyze the difference between groups. Statistical inferences were two‐sided and *P* value less than 0.05 was considered statistically significant.

## 4 RESULTS

Demographic characteristics of the study population were shown in Table 1. The study population included 1351 (49.6%) never smokers, 957 (35.1%) former smokers, 180 (6.6%) occasional smokers, 106 (3.9%) smokers with less than 10 cigarettes per day, 131 (4.8%) smokers with no less than 10 cigarettes per day. Never smokers were slightly older than other groups (*P* < 0.001). Daily smokers (including smokers with less than 10 cigarettes per day and smokers with no less than 10 cigarettes per day) accounted for large percentage of lowest degree of BMI (*P* < 0.05) and the percentage of normal (18.5‐24 Kg/m^2^) in never smokers were higher relative to others (*P* < 0.05). Daily smokers had lower education background compared to other group, and never smokers had higher education level (*P* < 0.001). Considering marital and living status, daily smokers were more likely to get divorced/separated/widowed/single (*P* < 0.05) and they were more likely to live alone (*P* < 0.001). The percentage of never smokers in high level of personal income (≥3000) was higher than others (*P* < 0.05). In contrast, the percentage of smokers with not less than 10 cigarettes per day in low lever personal income was higher than the rest (*P* < 0.05). The mean duration of disease since 2013 for study population was 7.99 ± 6.88 years. No significant difference was found in terms of financial status, so was the number of chronic diseases they acquired among five groups.

**Table 1:** Baseline characteristics of 2725 study subjects according to self‐reported smoking status in 2013

Variable	Never smokers (n = 1351)	Former smokers (n = 957)	Current smokers (n = 417)			P Value
			Occasional smokers (n = 180)	<10 cigarettes/d (n = 106)	≥10 cigarettes/d (n = 131)	
Age in 2013, median (Q1‐Q3), y	66 (60‐72)	63 (59‐69)	62 (58‐67)	62 (59‐68)	61 (58‐66)	<0.0001
Body mass index, mean ± SD	23.10 ± 3.07	23.46 ± 3.19	23.42 ± 2.73	22.89 ± 3.22	23.10 ± 3.52	0.032
BMI, No. (%)						
<18.5	76 (5.63%)	49 (5.12%)	8 (4.44%)	12 (11.32%)	15 (11.45%)	0.0036
18.5‐24	773 (57.22%)	496 (51.83%)	89 (49.44%)	53 (50.00%)	60 (45.80%)	
24‐28	422 (31.24%)	340 (35.53%)	73 (40.56%)	35 (33.02%)	45 (34.35%)	
>28	80 (5.92%)	72 (7.52%)	10 (5.56%)	6 (5.66%)	11 (8.40%)	
Education						
Basic	548 (40.56%)	429 (44.83%)	74 (41.11%)	55 (51.89%)	74 (56.49%)	0.0004
Secondary	652 (48.26%)	457 (47.75%)	94 (52.22%)	45 (42.45%)	51 (38.93%)	
Higher	151 (11.18%)	71 (7.42%)	12 (6.67%)	6 (5.66%)	6 (4.58%)	
Marital status						
Married/cohabitation	1222 (90.45%)	895 (93.52%)	169 (93.89%)	94 (88.68%)	111 (84.73%)	0.0020
Divorced/separated/widowed/single	129 (9.55%)	62 (6.48%)	11 (6.11%)	12 (11.32%)	20 (15.27%)	
Living status						
With spouse	778 (57.59%)	483 (50.47%)	100 (55.56%)	51 (48.11%)	52 (39.69%)	<0.0001
With family	100 (7.40%)	57 (5.96%)	10 (5.56%)	8 (7.55%)	17 (12.98%)	
With spouse and family	397 (29.39%)	382 (39.92%)	66 (36.67%)	40 (37.74%)	52 (39.69%)	
Living alone	76 (5.63%)	35 (3.66%)	4 (2.22%)	7 (6.60%)	10 (7.63%)	
Family income per month						
Less than 1000	102 (7.55%)	88 (9.20%)	13 (7.22%)	9 (8.49%)	17 (12.98%)	0.1372
1001‐2000	196 (14.51%)	152 (15.88%)	22 (12.22%)	24 (22.64%)	27 (20.61%)	
2001‐3000	533 (39.45%)	380 (39.71%)	78 (43.33%)	41 (38.68%)	46 (35.11%)	
3001‐4000	241 (17.84%)	160 (16.72%)	35 (19.44%)	15 (14.15%)	25 (19.08%)	
More than 4000	279 (20.65%)	177 (18.50%)	32 (17.78%)	17 (16.04%)	16 (12.21%)	
Personal income						
Less than 1000	130 (9.62%)	112 (11.70%)	12 (6.67%)	11 (10.38%)	22 (16.79%)	0.0019
1000‐3000	829 (61.36%)	564 (58.93%)	124 (68.89%)	75 (70.75%)	86 (65.65%)	
More than 3000	392 (29.02%)	281 (29.36%)	44 (24.44%)	20 (18.87%)	23 (17.56%)	
Ill time since 2013	6.64 (3.79‐12.32)	4.73 (2.50‐9.08)	5.76 (3.09‐9.88)	5.89 (3.66‐10.74)	7.34 (4.22‐13.57)	<0.0001
Number of chronic diseases						
No	310 (22.95%)	176 (18.39%)	40 (22.22%)	22 (20.75%)	35 (26.72%)	0.0524
Yes	1041 (77.05%)	781 (81.61%)	140 (77.78%)	84 (79.25%)	96 (73.28%)	

The influences of different smoking status on EORTC QLQ‐C30 scores were listed in Table 2 and Table 3. After adjusting for sociodemographic characteristics (age, BMI, marital status, residency, education level, financial status, chronic diseases, and treatment status), the scores on global health status, social, role, cognitive functioning, nausea/vomiting, pain, dyspnea, insomnia, appetite loss, and constipation among groups of never smokers, former smokers, and current smokers had statistical difference (*P* < 0.05). Current smokers were reported to have higher scores in social, role, cognitive functioning, and had lower scores in nausea/vomiting, pain, dyspnea, and insomnia (*P* < 0.05). Former smokers had high global health status and experienced less appetite loss and constipation (*P* < 0.05). No difference was found in physical, emotional functioning as well as fatigue, constipation, diarrhea, and financial difficulty (*P* > 0.05) among three groups. Considering about the dose of smoking, the scores were increased in functional subscales and decreased in symptom subscales with the increase of tobacco use, although few variables (role function and symptom of pain) had statistical significance. Additionally, compared with never smokers, former smokers had better in global health status, role functioning, cognitive functioning, and experienced less symptoms of nausea/vomiting, pain, and appetite loss. Occasional smokers had better in cognitive function. Smokers with less than 10 cigarettes per day got better scores in social and role functioning and suffered from less insomnia symptom. Also, smokers with not less than 10 cigarettes per day experienced better in social, role and cognitive functioning, and had less symptoms of nausea/vomiting and pain.

**Table 2:** Adjusted QOL scores by self‐reported smoking status in 2013 [Mean scores (95% CI)]

	Never‐smokers (n = 1351)	Former smokers (n = 957)	Current smokers (n = 417)	*P* value	Current smokers			
					Occasional smokers (n = 180)	<10 cigarettes/d (n = 106)	≥10 cigarettes/d (n = 131)	*P* value
QL	62.18 (60.76, 63.61)	64.92 (63.29, 66.56)	64.33 (61.82, 66.84)	0.0391	64.17 (60.49, 67.85)	63.90 (59.16, 68.60)	66.93 (62.50, 71.38)	0.5702
PF	82.94 (82.13, 83.76)	83.16 (82.20, 84.12)	84.46 (83.00, 85.92)	0.2019	84.67 (82.57, 86.78)	84.26 (81.52, 87.01)	85.92 (83.45, 88.42)	0.6429
EF	85.95 (85.06, 86.84)	86.74 (85.68, 87.80)	86.53 (84.92, 88.13)	0.5184	84.88 (82.46, 87.29)	86.70 (83.57, 89.83)	87.61 (84.77, 90.47)	0.3443
SF	77.03 (75.81, 78.25)	76.66 (75.22, 78.11)	80.70 (78.51, 82.89)	0.0066	78.90 (75.73, 82.07)	80.65 (76.56, 84.74)	81.44 (77.71, 85.17)	0.5825
RF	88.67 (87.67, 89.66)	90.87 (89.69, 92.05)	91.27 (89.49, 93.06)	0.0052	88.75 (86.32, 91.18)	93.39 (90.26, 96.52)	93.71 (90.85, 96.57)	0.0158
CF	78.68 (77.68, 79.68)	80.57 (79.38, 81.75)	81.98 (80.20, 83.78)	0.0027	81.94 (79.23, 84.64)	81.80 (78.23, 85.36)	82.20 (79.00, 85.41)	0.9165
FA	26.66 (25.63, 27.68)	27.50 (26.29, 28.70)	26.96 (25.14, 28.79)	0.5899	27.12 (24.14, 30.12)	26.70 (22.84 30.57)	26.10 (22.55, 29.64)	0.9128
NV	4.75 (4.10, 5.40)	3.57 (2.80, 4.34)	3.37 (2.21, 4.54)	0.0305	4.62 (3.06, 6.20)	3.76 (1.74, 5.80)	2.07 (0.23, 3.91)	0.1228
PA	16.66 (15.64, 17.67)	14.97 (13.77, 16.17)	13.95 (12.13, 15.77)	0.0164	16.30 (13.66, 18.95)	13.63 (10.21, 17.05)	10.68 (7.54, 13.82)	0.0307
DY	15.39 (14.25, 16.53)	17.22 (15.88, 18.57)	14.05 (12.03, 16.07)	0.0216	14.60 (11.74, 17.46)	13.59 (9.90, 17.28)	12.80 (9.40, 16.21)	0.7328
SL	17.91 (16.69, 19.14)	15.48 (14.03, 16.93)	15.21 (13.02, 17.40)	0.0173	17.28 (13.98, 20.58)	11.32 (6.96, 15.67)	15.41 (11.48, 19.33)	0.1240
AP	10.60 (9.61, 11.58)	8.73 (7.57, 9.89)	10.11 (8.36, 11.87)	0.001	10.60 (7.69, 13.53)	11.33 (7.56, 15.10)	8.77 (5.32, 12.21)	0.5812
CO	11.87 (10.79, 12.96)	10.70 (9.41, 11.99)	12.06 (10.12, 14.02)	<0.001	13.27 (10.25, 16.29)	10.22 (6.32, 14.13)	11.33 (7.74, 14.92)	0.4564
DI	9.52 (8.56, 10.47)	9.34 (8.21, 10.46)	9.32 (7.63, 11.01)	0.9654	9.15 (6.51, 11.78)	7.92 (4.52, 11.34)	10.97 (7.86, 14.08)	0.4203
FI	29.83 (28.27, 31.39)	30.17 (28.33, 32.00)	30.12 (27.34, 32.90)	0.9608	33.38 (29.05, 37.70)	31.96 (26.38, 37.55)	30.76 (25.68, 35.85)	0.7475

Standard least squares regression was conducted controlling for age, BMI, marital status, residency, education level, financial status, chronic diseases, and treatment status.

CI, confidence interval; EORTC QLQ‐C30, the European Organization for the Research and Treatment of Cancer; QL, Global health status/QoL; PF, Physical Functioning; EF, Emotional Functioning; SF, Social Functioning; RF, Role Functioning; CF, Cognitive Functioning; FA, Fatigue; NV, Nausea/Vomiting; PA, Pain; DY, Dyspnea; SL, Insomnia; AP, Appetite loss; CO, Constipation; DI, Diarrhea; FI, Financial problems.

**Table 3:** Mean score differences among current and former smokers (adjusted according to sociodemographic characteristics) [Mean scores (95% CI)]

	Former smokers	Occasional smokers	<10 cigarettes/d	≥10 cigarettes/d
QL	2.74 (0.56, 4.92)*	1.00 (−3.04, 5.56)	1.61 (−3.48, 6.71)	4.30 (−0.49, 9.10)
PF	0.22 (−1.56, 1.49)	1.09 (−1.26, 3.44)	0.90 (−2.12, 3.92)	2.62 (−0.12, 5.37)
EF	0.79 (−0.60, 2.18)	−1.22 (−3.80, 1.36)	1.35 (−1.95, 4.64)	2.51 (−0.49, 5.51)
SF	−0.37 (−2.28, 1.54)	1.58 (−1.95, 5.12)	4.67 (0.16, 9.18)*	5.79 (1.68, 9.90)*
RF	2.20 (0.65, 3.76)*	−0.36 (−3.24, 2.52)	4.60 (0.94, 8.26)*	5.14 (1.79, 8.48)*
CF	1.89 (0.32, 3.45)*	3.26 (0.36, 6.15)*	3.12 (−0.89, 6.82)	3.52 (0.16, 6.89)*
FA	0.84 (−0.76, 2.43)	1.00 (−1.95, 3.94)	0.17 (−3.58, 3.94)	−0.58 (−4.02, 2.86)
NV	−1.18 (−2.20, −0.16)*	−0.35 (−2.23, 1.52)	−1.22 (−3.63,1.20)	−2.97 (−5.16, −0.77)*
PA	−1.69 (−3.27, −0.10)*	−0.09 (−3.02, 2.83)	−3.02 (−6.75, 0.71)	−6.18 (−9.60, −2.76)*
DY	1.83 (−0.06, 3.11)	−0.45 (−3.71, 2.82)	−1.63 (−5.77, 2.51)	−2.36 (−6.18, 1.46)
SL	−2.43 (−4.32, −0.52)	−0.63 (−4.15, 2.89)	−6.60 (−11.13, −2.07)*	−2.51 (−6.63, 1.62)
AP	−1.87 (−3.41, −0.33)*	0.12 (−2.72, 2.96)	0.56 (−3.06, 4.18)	−2.20 (−5.50, 1.11)
CO	−1.18 (−2.87, 0.52)	1.98 (−1.15, 5.12)	−1.79 (−5.78,2.21)	−0.73 (−5.40, 3.93)
DI	−0.18 (−1.67, 1.30)	−0.31 (−3.04, 2.43)	−1.66 (−5.15,1.83)	1.18 (−2.01, 4.36)
FI	0.33 (−2.10, 2.77)	2.31 (−2.18, 6.80)	−0.42 (−6.15, 5.32)	−1.98 (−7.20,3.24)

* *P* < 0.05; Standard least squares regression were conducted controlling for age, BMI, marital status, residency, education level, financial status, chronic diseases, and treatment status.

**Figure 1:**
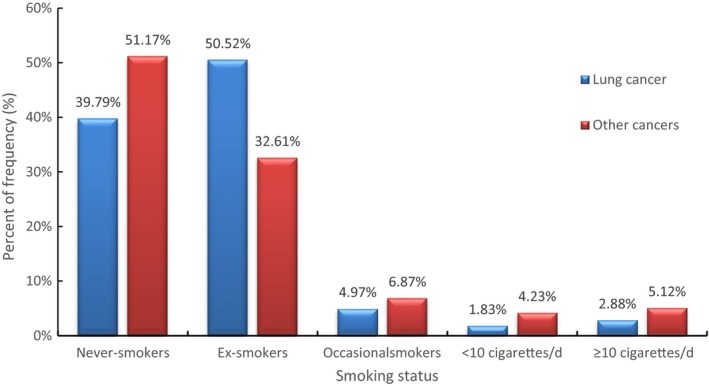
Smoking status between lung cancer and other cancers. *Notes*. Other cancers including types of colorectal cancer, gastric cancer, liver cancer, nasopharyngeal carcinoma, renal carcinoma, bladder cancer, prostatic carcinoma, laryngeal cancer, lymphoma, esophageal cancer, leukemia, thyroid cancer, and some rare cancers.

In Figure 1, the percentage of lung cancer in the group of never smokers, former smokers, occasional smokers, smokers with less than 10 cigarettes per day, and smokers with not less than 10 cigarettes per day were 39.79%, 50.52%, 4.97%, 1.83%, and 2.88%, respectively. Meanwhile other cancers accounted for 51.17%, 32.61%, 6.87%, 4.23%, and 5.12% in the same order among five groups, respectively. Lung cancer had higher percentage relative to other cancers in group of former smokers and accounted lower percentage compared to other cancers in other groups.

## 5 DISCUSSION

In our study, we evaluated QOL in groups of never smokers, former smokers, occasional smokers, smokers with less than 10 cigarettes per day, and smokers with more than 10 cigarettes per day among cancer survivors from Shanghai Cancer Rehabilitation Club. It was revealed that present smokers had better QOL compared to never smokers and former smokers, especially in social, role, cognitive functioning and experienced milder nausea/vomiting, pain, sleeping disorders, and dyspnea. Unfortunately, it seems that these conclusions were not in line with most studies, which suggested that smoking had significantly negative influence on QOL between never smokers and continuous smokers.^20^, ^26^, ^27^ Actually we could not agree more with this and our results did not mean to deliver that smoking could improve patients’ QOL. Instead, it strongly reminded researchers and clinicians some obscured situations among smokers in Chinese cancer survivors.

Considering about baseline information of study population, continuous smokers were slightly younger than other groups. They accounted for a large percentage of lowest and highest degree of BMI and lower education background compared to other group. In addition, they experienced worse relationship with their spouses and more likely to get divorced, which indicated that the overall situation of current smokers were not good.

The reality maybe obscured by subjective feeling of an individual which led to misleading results because QOL is a multidimensional construct that reflects a person's self‐rated perception of his or her life in terms of aspects of health.^27^, ^28^, ^29^ The substances of smoke, specifically nicotine, have effect on psychology and spirit, and make people relax and induce pleasure.^30^ Cancer survivors generally have greater psychological stress and usually suffer from damaged functional capacity, impaired family and sexual relationships, strained work and activities, financial difficulty and much more challenges.^31^, ^32^, ^33^, ^34^ Therefore, in order to relieve themselves, smoking is the best way to cope with emotionally difficult, relieving pain symptom and is regarded as a reward from work or stressful events.^35^, ^36^ In addition, unlike other foreign countries, tobacco has special social and cultural functions in Chinese society under long‐established cultures and customs. In china, cigarettes are regarded as a tool for social interaction or usually offered to friends, guests, and respected individuals as presents.^37^ Considering about that, those smokers always report being happier and felt more socially acceptable. This is in line with our results that daily smokers got higher score in social function.

Aside from that, our study has made some comparisons between lung cancer and other cancers concerning cigarettes smoking. The proportion of lung cancer in the never‐smoking group was small and much higher than that of other cancer groups in the quit‐smoking group. We all know that smoking is the predominant risk for lung cancer and quitting smoking has several positive benefits in high‐risk population or lung cancer patients.^38^ In general, it demonstrated that the implementation of smoke cessation and educational strategies given by health educators or doctors were well done in lung cancer. However, sufficient work was little more than accomplished in types of other cancer. This warned us to actively carry out such efforts to discourage smoking in other types of cancers or general population.

Generally, continued smoking after cancer diagnosis may result in underlying negative effect on QOL of cancer survivors, although the scores were high among smokers. The misleading scores concealed reality because of the reflection of subjective feeling of participants, and smoking may bring “happiness” to them. That prompted a strong call for the implementation of a comprehensive tobacco‐control policy and specific public health educational strategies. Furthermore, quitters had better QOL suggesting that it was never too late to adopt these healthy behaviors. Moreover, the effective smoking cessation or educational work among lung cancer patients is equally essential to promote into other cancers. Meanwhile, there are some limitations need to be acknowledged. First, since our study is a cross‐sectional study, no conclusion on causality can be drawn from our results. Secondly, there is another possibility that those who had good QOL have no inclination to take actions to quit smoking and we believe that current smokers would have better QOL after smoking cessation.^28^, ^39^ In addition, smoking status is strongly influenced by cancer diagnosis, as well as stages of cancer and clinical treatment. The dose of smokers was not detailedly investigated in our study, and it is better to assess smoking dosage instead in the future study.
